# Supplementary material for: Dissemination Routes of Carbapenem and Pan-Aminoglycoside Resistance Mechanisms in Hospital and Urban Wastewater Canalizations of Ghana
Source: mSystems. 2022 Feb 1;7(1):e01019-21. doi: 10.1128/msystems.01019-21 (PMC8805638; doi:10.1128/msystems.01019-21)
Supplement: TABLE S1 [file msystems.01019-21-st001.docx]

**Table S1**

| Sampling location | Sampling point | Wastewater canalization type | Number of samples | Latitude | Longitude |
| --- | --- | --- | --- | --- | --- |
| Tamale West Hospital  (TWH) | TWH-1 | Community (before the hospital) | 2 | 9.402198 | -0.850864 |
|  | TWH-2 | Hospital (drainage of the hospital) | 2 | 9.400990 | -0.850922 |
|  | TWH-3 | Community (after the hospital) | 2 | 9.404507 | -0.850758 |
| Tamale Central Hospital  (TCH) | TCH-1 | Community (before the hospital) | 2 | 9.407124 | -0.837020 |
|  | TCH-2 | Hospital (drainage of the hospital) | 2 | 9.407126 | -0.837348 |
|  | TCH-3 | Community (after the hospital) | 2 | 9.407137 | -0.837020 |
| Tamale Teaching Hospital  (TTH) | TTH-1 | Community (before the hospital) | 2 | 9.394171 | -0.822951 |
|  | TTH-2 | Hospital (drainage of the hospital) | 2 | 9.393920 | -0.822843 |
|  | TTH-3 | Community (after the hospital) | 2 | 9.392537 | -0.819811 |
| Urban Waste Treatment Plant  (UWTP) | UWTP-1 | First treatment pond | 2 | 9.442864 | -0.756785 |
|  | UWTP-2 | Last tretament pond | 2 | 9.444945 | -0.757774 |
|  | UWTP-3 | Drainage of the UWTP | 2 | 9.441993 | -0.752466 |
