# Supplementary figures and images for: Dissemination Routes of Carbapenem and Pan-Aminoglycoside Resistance Mechanisms in Hospital and Urban Wastewater Canalizations of Ghana
Source: mSystems. 2022 Feb 1;7(1):e01019-21. doi: 10.1128/msystems.01019-21 (PMC8805638; doi:10.1128/msystems.01019-21)

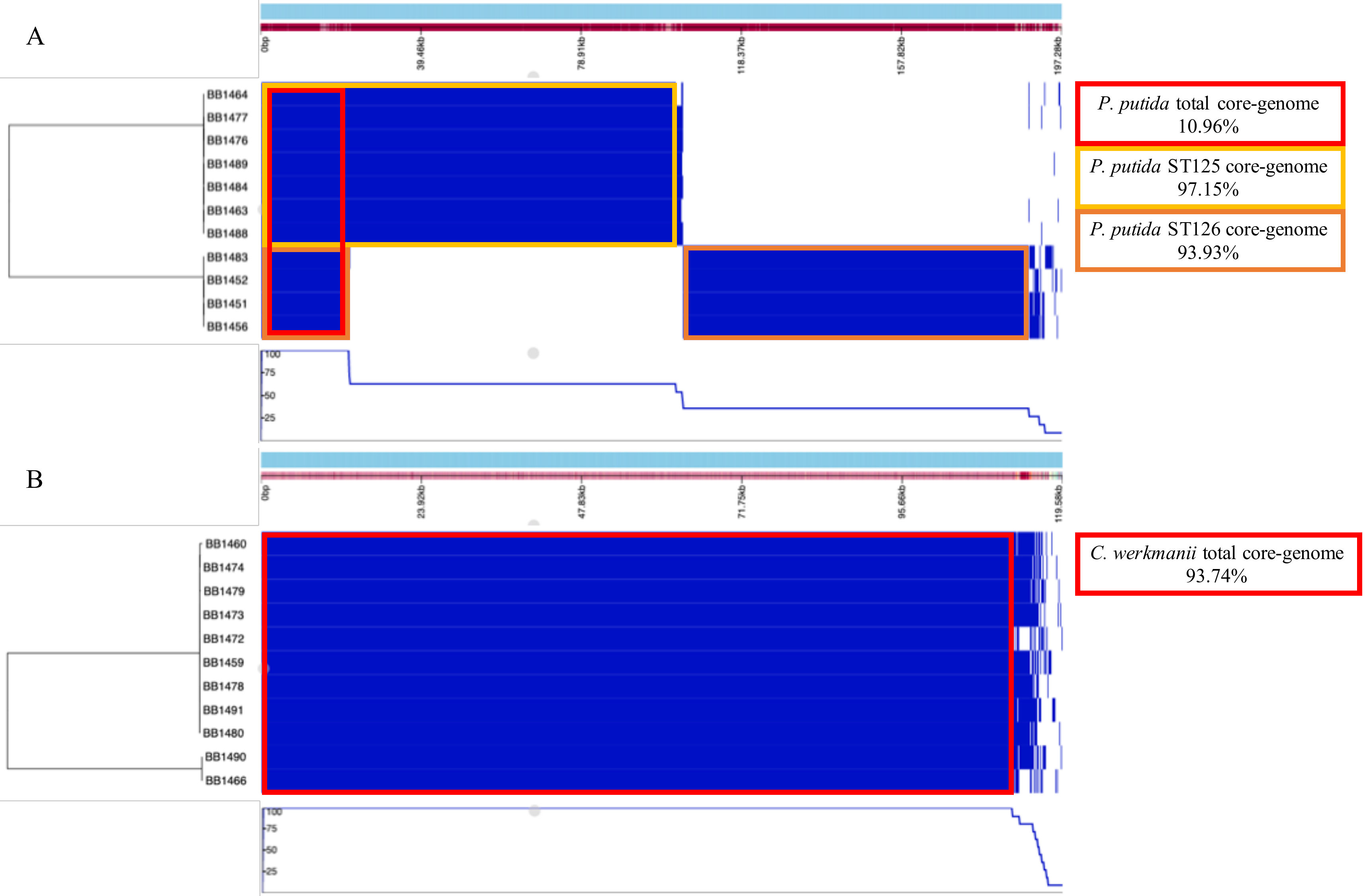

Supplement: FIG S1 [file msystems.01019-21-sf001.tif]

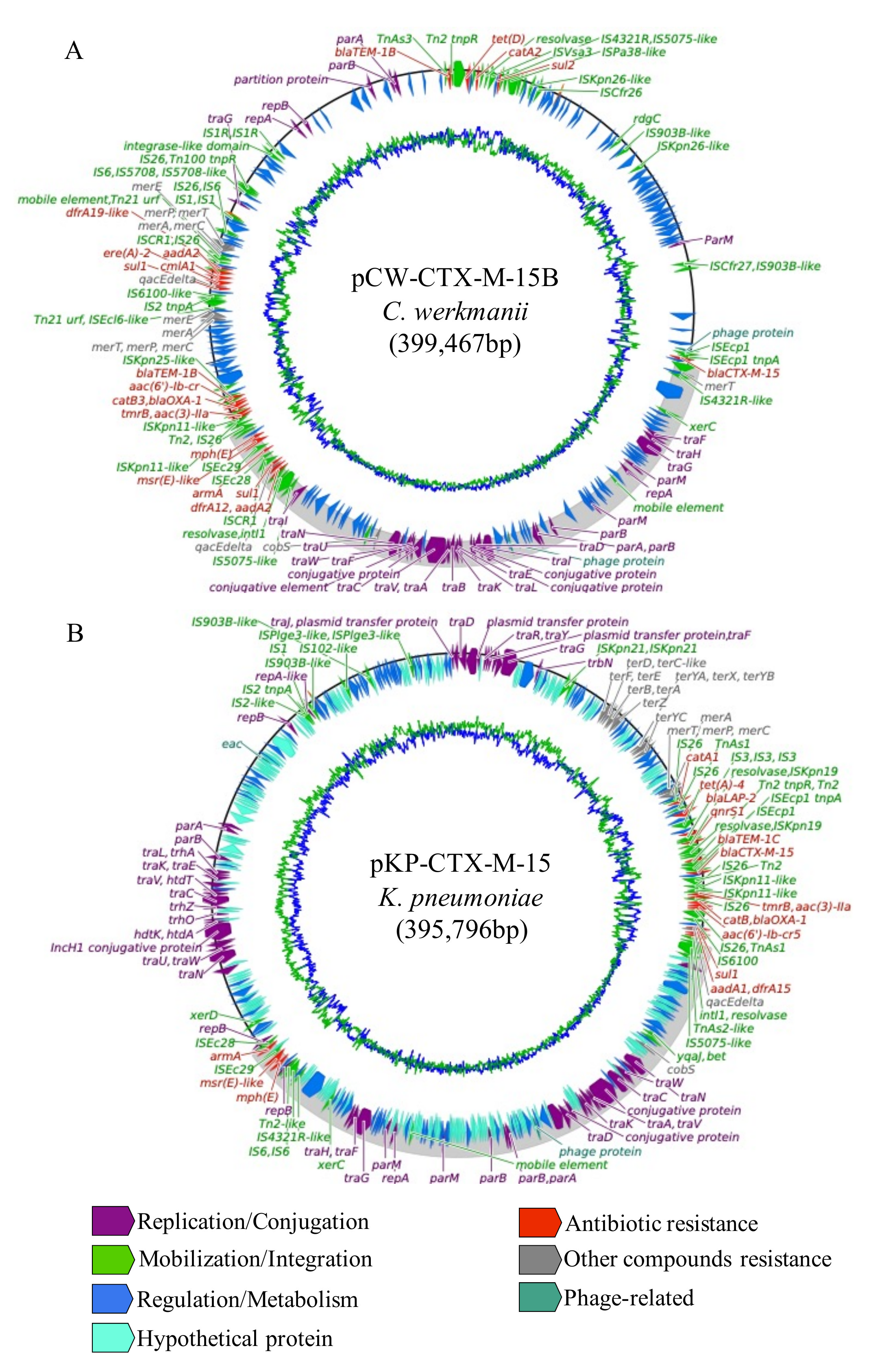

Supplement: FIG S4 [file msystems.01019-21-sf004.tif]
