# Supplementary material for: Dissemination Routes of Carbapenem and Pan-Aminoglycoside Resistance Mechanisms in Hospital and Urban Wastewater Canalizations of Ghana
Source: mSystems. 2022 Feb 1;7(1):e01019-21. doi: 10.1128/msystems.01019-21 (PMC8805638; doi:10.1128/msystems.01019-21)
Supplement: TABLE S2 [file msystems.01019-21-st002.docx]

**Table S2**

| Isolate | ENA sample | Species | Source | Latitude | Longitude | ENA project | Sequencing technology | ENA run | Total sequences | Mean lenght (bp) | %GC | Coverage (X) |
| --- | --- | --- | --- | --- | --- | --- | --- | --- | --- | --- | --- | --- |
| BB1451 | ERS4590898 | *Pseudomonas putida* | TCH-2 | 9.407126 | -0.837348 | PRJEB38443 | Nanopore | ERR4184085 | 181,038 | 5,234.60 | 60.90 | 157.90 |
| BB1453 | ERS4590900 | *Providencia rettgeri* | TCH-2 | 9.407126 | -0.837348 | PRJEB38443 | Nanopore | ERR4184136 | 317,311 | 7,161.73 | 41.29 | 444.56 |
| BB1454 | ERS4590901 | *Comamonas aquatica* | TCH-2 | 9.407126 | -0.837348 | PRJEB38443 | Nanopore | ERR4184149 | 233,983 | 6,648.26 | 62.99 | 383.69 |
| BB1455 | ERS4590902 | *Delftia tsuruhatensis* | TCH-2 | 9.407126 | -0.837348 | PRJEB38443 | Nanopore | ERR4184150 | 120,437 | 7,892.12 | 65.39 | 154.91 |
| BB1456 | ERS4590903 | *Pseudomonas putida* | TCH-3 | 9.407137 | -0.83702 | PRJEB38443 | Nanopore | ERR4184151 | 45,062 | 7,247.55 | 60.75 | 54.43 |
| BB1459 | ERS4590906 | *Citrobacter werkmanii* | TCH-3 | 9.407137 | -0.83702 | PRJEB38443 | Nanopore | ERR4184152 | 120,011 | 2,621.89 | 51.52 | 51.37 |
| BB1460 | ERS4590907 | *Citrobacter werkmanii* | TCH-3 | 9.407137 | -0.83702 | PRJEB38443 | Nanopore | ERR4184154 | 213,329 | 2,210.29 | 51.68 | 76.59 |
| BB1491 | ERS4590908 | *Citrobacter werkmanii* | TCH-3 | 9.407137 | -0.83702 | PRJEB38443 | Nanopore | ERR4184156 | 301,843 | 6,031.78 | 51.43 | 295.68 |
| BB1462 | ERS4590909 | *Pseudomonas stutzeri* | TTH-1 | 9.394171 | -0.822951 | PRJEB38443 | Nanopore | ERR4183543 | 75,370 | 8,431.19 | 62.22 | 139.58 |
| BB1465 | ERS4590912 | *Klebsiella pneumoniae* | TTH-1 | 9.394171 | -0.822951 | PRJEB38443 | Nanopore | ERR4183544 | 266,558 | 6,162.82 | 55.80 | 273.69 |
| BB1466 | ERS4590913 | *Citrobacter werkmanii* | TTH-1 | 9.394171 | -0.822951 | PRJEB38443 | Nanopore | ERR4183589 | 271,513 | 5,914.87 | 51.46 | 264.27 |
| BB1467 | ERS4590914 | *Providencia rettgeri* | TTH-1 | 9.394171 | -0.822951 | PRJEB38443 | Nanopore | ERR4183608 | 177,391 | 7,930.50 | 40.92 | 311.36 |
| BB1468 | ERS4590915 | *Citrobacter youngae* | TTH-1 | 9.394171 | -0.822951 | PRJEB38443 | Nanopore | ERR4184095 | 334,432 | 5,200.30 | 50.95 | 316.07 |
| BB1471 | ERS4590918 | *Escherichia coli* | TTH-2 | 9.39392 | -0.822843 | PRJEB38443 | Nanopore | ERR4184096 | 67,216 | 7,209.44 | 50.56 | 86.24 |
| BB1472 | ERS4590919 | *Citrobacter werkmanii* | TTH-2 | 9.39392 | -0.822843 | PRJEB38443 | Nanopore | ERR4184097 | 253,283 | 4,781.89 | 51.42 | 199.43 |
| BB1473 | ERS4590920 | *Citrobacter werkmanii* | TTH-2 | 9.39392 | -0.822843 | PRJEB38443 | Nanopore | ERR4184098 | 274,462 | 6,325.17 | 51.46 | 282.69 |
| BB1474 | ERS4590921 | *Citrobacter werkmanii* | TTH-2 | 9.39392 | -0.822843 | PRJEB38443 | Nanopore | ERR4184099 | 350,020 | 5,582.37 | 51.40 | 317.96 |
| BB1475 | ERS4590922 | *Pseudomonas aeruginosa* | TTH-3 | 9.392537 | -0.819811 | PRJEB38443 | Nanopore | ERR4184100 | 167,396 | 3,550.19 | 63.86 | 84.26 |
| BB1478 | ERS4590925 | *Citrobacter werkmanii* | TTH-3 | 9.392537 | -0.819811 | PRJEB38443 | Nanopore | ERR4184108 | 343,234 | 6,270.24 | 51.49 | 351.91 |
| BB1479 | ERS4590926 | *Citrobacter werkmanii* | TTH-3 | 9.392537 | -0.819811 | PRJEB38443 | Nanopore | ERR4184109 | 439,923 | 5,555.58 | 51.39 | 398.35 |
| BB1480 | ERS4590927 | *Citrobacter werkmanii* | TTH-3 | 9.392537 | -0.819811 | PRJEB38443 | Nanopore | ERR4184110 | 383,836 | 5,098.69 | 51.44 | 318.59 |
| BB1484 | ERS4590931 | *Pseudomonas putida* | TWH-2 | 9.40099 | -0.850922 | PRJEB38443 | Nanopore | ERR4184111 | 157,987 | 5,021.85 | 60.91 | 139.20 |
| BB1486 | ERS4590933 | *Comamonas aquatica* | TWH-2 | 9.40099 | -0.850922 | PRJEB38443 | Nanopore | ERR4184112 | 1,146,027 | 2,780.84 | 63.19 | 756.32 |
| BB1487 | ERS4590934 | *Providencia rettgeri* | TWH-2 | 9.40099 | -0.850922 | PRJEB38443 | Nanopore | ERR4184131 | 297,297 | 8,057.61 | 41.34 | 474.85 |
| BB1490 | ERS4590937 | *Citrobacter werkmanii* | UWTP-2 | 9.444945 | -0.757774 | PRJEB38443 | Nanopore | ERR4184205 | 351,044 | 6,035.70 | 51.44 | 343.99 |
