# Supplementary material for: Dissemination Routes of Carbapenem and Pan-Aminoglycoside Resistance Mechanisms in Hospital and Urban Wastewater Canalizations of Ghana
Source: mSystems. 2022 Feb 1;7(1):e01019-21. doi: 10.1128/msystems.01019-21 (PMC8805638; doi:10.1128/msystems.01019-21)
Supplement: TABLE S3 [file msystems.01019-21-st003.docx]

**Table S3**

| Isolate | ENA sample | Species | Source | Latitude | Longitude | ENA project | Sequencing technology | ENA run | Total reads | Lenght (bp) | %GC | Coverage (X) |
| --- | --- | --- | --- | --- | --- | --- | --- | --- | --- | --- | --- | --- |
| BB1451 | ERS4590898 | *Pseudomonas putida* | TCH-2 | 9.407126 | -0.837348 | PRJEB38442 | Illumina | ERR4180949 | 932,684 | 300 | 62.00 | 47.08 |
| BB1452 | ERS4590899 | *Pseudomonas putida* | TCH-2 | 9.407126 | -0.837348 | PRJEB38442 | Illumina | ERR4181068 | 1,017,302 | 300 | 62.00 | 51.83 |
| BB1453 | ERS4590900 | *Providencia rettgeri* | TCH-2 | 9.407126 | -0.837348 | PRJEB38442 | Illumina | ERR4181071 | 2,335,482 | 300 | 42.00 | 141.18 |
| BB1454 | ERS4590901 | *Comamonas aquatica* | TCH-2 | 9.407126 | -0.837348 | PRJEB38442 | Illumina | ERR4181091 | 1,843,716 | 300 | 65.00 | 138.84 |
| BB1455 | ERS4590902 | *Delftia tsuruhatensis* | TCH-2 | 9.407126 | -0.837348 | PRJEB38442 | Illumina | ERR4181072 | 2,513,458 | 300 | 67.00 | 123.46 |
| BB1456 | ERS4590903 | *Pseudomonas putida* | TCH-3 | 9.407137 | -0.83702 | PRJEB38442 | Illumina | ERR4181092 | 1,659,690 | 300 | 62.00 | 83.77 |
| BB1457 | ERS4590904 | *Aeromonas hydrophila* | TCH-3 | 9.407137 | -0.83702 | PRJEB38442 | Illumina | ERR4181123 | 816,670 | 300 | 61.00 | 50.00 |
| BB1458 | ERS4590905 | *Pseudomonas aeruginosa* | TCH-3 | 9.407137 | -0.83702 | PRJEB38442 | Illumina | ERR4181124 | 1,434,066 | 300 | 65.00 | 62.32 |
| BB1459 | ERS4590906 | *Citrobacter werkmanii* | TCH-3 | 9.407137 | -0.83702 | PRJEB38442 | Illumina | ERR4181087 | 1,500,392 | 300 | 52.00 | 75.81 |
| BB1460 | ERS4590907 | *Citrobacter werkmanii* | TCH-3 | 9.407137 | -0.83702 | PRJEB38442 | Illumina | ERR4180915 | 880,114 | 300 | 52.00 | 44.51 |
| BB1491 | ERS4590908 | *Citrobacter werkmanii* | TCH-3 | 9.407137 | -0.83702 | PRJEB38442 | Illumina | ERR4180923 | 1,164,554 | 300 | 52.00 | 58.96 |
| BB1462 | ERS4590909 | *Pseudomonas stutzeri* | TTH-1 | 9.394171 | -0.822951 | PRJEB38442 | Illumina | ERR4180924 | 689,192 | 300 | 63.00 | 45.66 |
| BB1463 | ERS4590910 | *Pseudomonas putida* | TTH-1 | 9.394171 | -0.822951 | PRJEB38442 | Illumina | ERR4181088 | 830,110 | 300 | 62.00 | 44.01 |
| BB1464 | ERS4590911 | *Pseudomonas putida* | TTH-1 | 9.394171 | -0.822951 | PRJEB38442 | Illumina | ERR4180929 | 1,256,504 | 300 | 62.00 | 67.19 |
| BB1465 | ERS4590912 | *Klebsiella pneumoniae* | TTH-1 | 9.394171 | -0.822951 | PRJEB38442 | Illumina | ERR4180935 | 861,928 | 300 | 56.00 | 43.95 |
| BB1466 | ERS4590913 | *Citrobacter werkmanii* | TTH-1 | 9.394171 | -0.822951 | PRJEB38442 | Illumina | ERR4180938 | 1,070,420 | 300 | 52.00 | 54.70 |
| BB1467 | ERS4590914 | *Providencia rettgeri* | TTH-1 | 9.394171 | -0.822951 | PRJEB38442 | Illumina | ERR4180939 | 1,794,408 | 300 | 42.00 | 119.71 |
| BB1468 | ERS4590915 | *Citrobacter youngae* | TTH-1 | 9.394171 | -0.822951 | PRJEB38442 | Illumina | ERR4181125 | 722,566 | 300 | 51.00 | 40.49 |
| BB1470 | ERS4590917 | *Pseudomonas stutzeri* | TTH-2 | 9.39392 | -0.822843 | PRJEB38442 | Illumina | ERR4180941 | 1,802,706 | 300 | 63.00 | 119.78 |
| BB1471 | ERS4590918 | *Escherichia coli* | TTH-2 | 9.39392 | -0.822843 | PRJEB38442 | Illumina | ERR4181089 | 297,874 | 300 | 51.00 | 16.52 |
| BB1472 | ERS4590919 | *Citrobacter werkmanii* | TTH-2 | 9.39392 | -0.822843 | PRJEB38442 | Illumina | ERR4180942 | 765,074 | 300 | 52.00 | 39.27 |
| BB1473 | ERS4590920 | *Citrobacter werkmanii* | TTH-2 | 9.39392 | -0.822843 | PRJEB38442 | Illumina | ERR4180943 | 1,356,908 | 300 | 52.00 | 68.78 |
| BB1474 | ERS4590921 | *Citrobacter werkmanii* | TTH-2 | 9.39392 | -0.822843 | PRJEB38442 | Illumina | ERR4180944 | 700,108 | 300 | 52.00 | 35.51 |
| BB1475 | ERS4590922 | *Pseudomonas aeruginosa* | TTH-3 | 9.392537 | -0.819811 | PRJEB38442 | Illumina | ERR4180945 | 675,050 | 300 | 65.00 | 29.91 |
| BB1476 | ERS4590923 | *Pseudomonas putida* | TTH-3 | 9.392537 | -0.819811 | PRJEB38442 | Illumina | ERR4180946 | 1,576,404 | 300 | 62.00 | 83.90 |
| BB1477 | ERS4590924 | *Pseudomonas putida* | TTH-3 | 9.392537 | -0.819811 | PRJEB38442 | Illumina | ERR4180948 | 917,670 | 300 | 62.00 | 48.75 |
| BB1478 | ERS4590925 | *Citrobacter werkmanii* | TTH-3 | 9.392537 | -0.819811 | PRJEB38442 | Illumina | ERR4181090 | 1,458,538 | 300 | 52.00 | 73.90 |
| BB1479 | ERS4590926 | *Citrobacter werkmanii* | TTH-3 | 9.392537 | -0.819811 | PRJEB38442 | Illumina | ERR4180950 | 626,354 | 300 | 52.00 | 31.73 |
| BB1480 | ERS4590927 | *Citrobacter werkmanii* | TTH-3 | 9.392537 | -0.819811 | PRJEB38442 | Illumina | ERR4180951 | 834,020 | 300 | 52.00 | 42.27 |
| BB1483 | ERS4590930 | *Pseudomonas putida* | TWH-1 | 9.402198 | -0.850864 | PRJEB38442 | Illumina | ERR4181062 | 1,144,248 | 300 | 62.00 | 57.51 |
| BB1484 | ERS4590931 | *Pseudomonas putida* | TWH-2 | 9.40099 | -0.850922 | PRJEB38442 | Illumina | ERR4181063 | 2,041,776 | 300 | 62.00 | 108.63 |
| BB1486 | ERS4590933 | *Comamonas aquatica* | TWH-2 | 9.40099 | -0.850922 | PRJEB38442 | Illumina | ERR4181065 | 1,953,000 | 300 | 64.00 | 146.61 |
| BB1487 | ERS4590934 | *Providencia rettgeri* | TWH-2 | 9.40099 | -0.850922 | PRJEB38442 | Illumina | ERR4181066 | 1,928,686 | 300 | 43.00 | 117.56 |
| BB1488 | ERS4590935 | *Pseudomonas putida* | TWH-3 | 9.404507 | -0.850758 | PRJEB38442 | Illumina | ERR4181067 | 2,160,166 | 300 | 62.00 | 114.99 |
| BB1489 | ERS4590936 | *Pseudomonas putida* | TWH-3 | 9.404507 | -0.850758 | PRJEB38442 | Illumina | ERR4181069 | 851,226 | 300 | 62.00 | 45.26 |
| BB1490 | ERS4590937 | *Citrobacter werkmanii* | UWTP-2 | 9.444945 | -0.757774 | PRJEB38442 | Illumina | ERR4181070 | 572,664 | 300 | 52.00 | 29.04 |
