# Supplementary material for: Dissemination Routes of Carbapenem and Pan-Aminoglycoside Resistance Mechanisms in Hospital and Urban Wastewater Canalizations of Ghana
Source: mSystems. 2022 Feb 1;7(1):e01019-21. doi: 10.1128/msystems.01019-21 (PMC8805638; doi:10.1128/msystems.01019-21)
Supplement: TABLE S4 [file msystems.01019-21-st004.docx]

**Table S4**

| Isolate | ENA sample | Species | Source | Latitude | Longitude | ENA project | Assembler | ENA analysis | Total contigs | Total length (bp) | %GC | N50 (bp) |
| --- | --- | --- | --- | --- | --- | --- | --- | --- | --- | --- | --- | --- |
| BB1451 | ERS4590898 | *Pseudomonas putida* | TCH-2 | 9.407126 | -0.837348 | PRJEB38442 | SPAdes | ERZ1396001 | 148 | 5,943,262 | 62.54 | 115,332 |
| BB1452 | ERS4590899 | *Pseudomonas putida* | TCH-2 | 9.407126 | -0.837348 | PRJEB38442 | SPAdes | ERZ1396012 | 141 | 5,888,045 | 62.58 | 131,773 |
| BB1453 | ERS4590900 | *Providencia rettgeri* | TCH-2 | 9.407126 | -0.837348 | PRJEB38442 | SPAdes | ERZ1396015 | 86 | 4,962,592 | 40.72 | 273,557 |
| BB1454 | ERS4590901 | *Comamonas aquatica* | TCH-2 | 9.407126 | -0.837348 | PRJEB38442 | SPAdes | ERZ1396016 | 139 | 3,983,851 | 64.84 | 59,627 |
| BB1455 | ERS4590902 | *Delftia tsuruhatensis* | TCH-2 | 9.407126 | -0.837348 | PRJEB38442 | SPAdes | ERZ1396017 | 102 | 6,107,679 | 67.23 | 148,859 |
| BB1456 | ERS4590903 | *Pseudomonas putida* | TCH-3 | 9.407137 | -0.83702 | PRJEB38442 | SPAdes | ERZ1396018 | 104 | 5,943,430 | 62.55 | 142,881 |
| BB1457 | ERS4590904 | *Aeromonas hydrophila* | TCH-3 | 9.407137 | -0.83702 | PRJEB38442 | SPAdes | ERZ1396019 | 126 | 4,899,768 | 61.58 | 88,788 |
| BB1458 | ERS4590905 | *Pseudomonas aeruginosa* | TCH-3 | 9.407137 | -0.83702 | PRJEB38442 | SPAdes | ERZ1396020 | 128 | 6,902,830 | 65.86 | 172,075 |
| BB1459 | ERS4590906 | *Citrobacter werkmanii* | TCH-3 | 9.407137 | -0.83702 | PRJEB38442 | SPAdes | ERZ1395981 | 190 | 5,937,078 | 51.76 | 186,701 |
| BB1460 | ERS4590907 | *Citrobacter werkmanii* | TCH-3 | 9.407137 | -0.83702 | PRJEB38442 | SPAdes | ERZ1395982 | 208 | 5,932,234 | 51.79 | 127,321 |
| BB1491 | ERS4590908 | *Citrobacter werkmanii* | TCH-3 | 9.407137 | -0.83702 | PRJEB38442 | SPAdes | ERZ1395983 | 205 | 5,925,810 | 51.8 | 133,071 |
| BB1462 | ERS4590909 | *Pseudomonas stutzeri* | TTH-1 | 9.394171 | -0.822951 | PRJEB38442 | SPAdes | ERZ1395984 | 65 | 4,528,623 | 63.86 | 376,522 |
| BB1463 | ERS4590910 | *Pseudomonas putida* | TTH-1 | 9.394171 | -0.822951 | PRJEB38442 | SPAdes | ERZ1395985 | 165 | 5,658,042 | 62.34 | 169,040 |
| BB1464 | ERS4590911 | *Pseudomonas putida* | TTH-1 | 9.394171 | -0.822951 | PRJEB38442 | SPAdes | ERZ1395986 | 113 | 5,610,240 | 62.46 | 232,708 |
| BB1465 | ERS4590912 | *Klebsiella pneumoniae* | TTH-1 | 9.394171 | -0.822951 | PRJEB38442 | SPAdes | ERZ1395987 | 186 | 5,884,055 | 56.55 | 139,437 |
| BB1466 | ERS4590913 | *Citrobacter werkmanii* | TTH-1 | 9.394171 | -0.822951 | PRJEB38442 | SPAdes | ERZ1395988 | 180 | 5,870,430 | 51.82 | 127,321 |
| BB1467 | ERS4590914 | *Providencia rettgeri* | TTH-1 | 9.394171 | -0.822951 | PRJEB38442 | SPAdes | ERZ1395989 | 160 | 4,496,904 | 40.4 | 72,270 |
| BB1468 | ERS4590915 | *Citrobacter youngae* | TTH-1 | 9.394171 | -0.822951 | PRJEB38442 | SPAdes | ERZ1395990 | 125 | 5,353,511 | 51.42 | 285,910 |
| BB1470 | ERS4590917 | *Pseudomonas stutzeri* | TTH-2 | 9.39392 | -0.822843 | PRJEB38442 | SPAdes | ERZ1395992 | 38 | 4,515,152 | 63.95 | 438,427 |
| BB1471 | ERS4590918 | *Escherichia coli* | TTH-2 | 9.39392 | -0.822843 | PRJEB38442 | SPAdes | ERZ1395993 | 498 | 5,410,232 | 50.97 | 28,104 |
| BB1472 | ERS4590919 | *Citrobacter werkmanii* | TTH-2 | 9.39392 | -0.822843 | PRJEB38442 | SPAdes | ERZ1395994 | 209 | 5,845,331 | 51.83 | 95,972 |
| BB1473 | ERS4590920 | *Citrobacter werkmanii* | TTH-2 | 9.39392 | -0.822843 | PRJEB38442 | SPAdes | ERZ1395995 | 158 | 5,918,475 | 51.82 | 151,154 |
| BB1474 | ERS4590921 | *Citrobacter werkmanii* | TTH-2 | 9.39392 | -0.822843 | PRJEB38442 | SPAdes | ERZ1395996 | 183 | 5,914,765 | 51.81 | 127,321 |
| BB1475 | ERS4590922 | *Pseudomonas aeruginosa* | TTH-3 | 9.392537 | -0.819811 | PRJEB38442 | SPAdes | ERZ1395997 | 125 | 6,770,939 | 66.1 | 191,171 |
| BB1476 | ERS4590923 | *Pseudomonas putida* | TTH-3 | 9.392537 | -0.819811 | PRJEB38442 | SPAdes | ERZ1395998 | 108 | 5,636,973 | 62.45 | 225,948 |
| BB1477 | ERS4590924 | *Pseudomonas putida* | TTH-3 | 9.392537 | -0.819811 | PRJEB38442 | SPAdes | ERZ1395999 | 139 | 5,647,647 | 62.39 | 209,778 |
| BB1478 | ERS4590925 | *Citrobacter werkmanii* | TTH-3 | 9.392537 | -0.819811 | PRJEB38442 | SPAdes | ERZ1396000 | 151 | 5,920,773 | 51.81 | 187,166 |
| BB1479 | ERS4590926 | *Citrobacter werkmanii* | TTH-3 | 9.392537 | -0.819811 | PRJEB38442 | SPAdes | ERZ1396002 | 194 | 5,921,401 | 51.81 | 118,755 |
| BB1480 | ERS4590927 | *Citrobacter werkmanii* | TTH-3 | 9.392537 | -0.819811 | PRJEB38442 | SPAdes | ERZ1396003 | 171 | 5,919,819 | 51.82 | 128,083 |
| BB1483 | ERS4590930 | *Pseudomonas putida* | TWH-1 | 9.402198 | -0.850864 | PRJEB38442 | SPAdes | ERZ1396006 | 107 | 5,969,288 | 62.53 | 133,755 |
| BB1484 | ERS4590931 | *Pseudomonas putida* | TWH-2 | 9.40099 | -0.850922 | PRJEB38442 | SPAdes | ERZ1396007 | 111 | 5,638,808 | 62.44 | 221,187 |
| BB1486 | ERS4590933 | *Comamonas aquatica* | TWH-2 | 9.40099 | -0.850922 | PRJEB38442 | SPAdes | ERZ1396009 | 129 | 3,996,415 | 64.65 | 57,058 |
| BB1487 | ERS4590934 | *Providencia rettgeri* | TWH-2 | 9.40099 | -0.850922 | PRJEB38442 | SPAdes | ERZ1396010 | 279 | 4,921,604 | 40.86 | 41,520 |
| BB1488 | ERS4590935 | *Pseudomonas putida* | TWH-3 | 9.404507 | -0.850758 | PRJEB38442 | SPAdes | ERZ1396011 | 113 | 5,635,895 | 62.46 | 232,708 |
| BB1489 | ERS4590936 | *Pseudomonas putida* | TWH-3 | 9.404507 | -0.850758 | PRJEB38442 | SPAdes | ERZ1396013 | 124 | 5,641,562 | 62.43 | 169,002 |
| BB1490 | ERS4590937 | *Citrobacter werkmanii* | UWTP-2 | 9.444945 | -0.757774 | PRJEB38442 | SPAdes | ERZ1396014 | 198 | 5,916,058 | 51.82 | 92,990 |
