# Supplementary material for: Dissemination Routes of Carbapenem and Pan-Aminoglycoside Resistance Mechanisms in Hospital and Urban Wastewater Canalizations of Ghana
Source: mSystems. 2022 Feb 1;7(1):e01019-21. doi: 10.1128/msystems.01019-21 (PMC8805638; doi:10.1128/msystems.01019-21)
Supplement: TABLE S5 [file msystems.01019-21-st005.docx]

**Table S5**

| Isolate | ENA sample | Species | Source | Latitude | Longitude | ENA project | Assembler | ENA analysis | Total contigs | Total length (bp) | %GC | N50 (bp) |
| --- | --- | --- | --- | --- | --- | --- | --- | --- | --- | --- | --- | --- |
| BB1451 | ERS4590898 | *Pseudomonas putida* | TCH-2 | 9.407126 | -0.837348 | PRJEB38443 | Unicycler | ERZ1426502 | 1 | 5,862,847 | 62.52 | 5,862,847 |
| BB1453 | ERS4590900 | *Providencia rettgeri* | TCH-2 | 9.407126 | -0.837348 | PRJEB38443 | Unicycler | ERZ1416196 | 5 | 4,991,120 | 40.83 | 4,666,977 |
| BB1454 | ERS4590901 | *Comamonas aquatica* | TCH-2 | 9.407126 | -0.837348 | PRJEB38443 | Unicycler | ERZ1426503 | 1 | 4,047,112 | 64.71 | 4,047,112 |
| BB1455 | ERS4590902 | *Delftia tsuruhatensis* | TCH-2 | 9.407126 | -0.837348 | PRJEB38443 | Unicycler | ERZ1426504 | 1 | 6,126,880 | 67.24 | 6,126,880 |
| BB1456 | ERS4590903 | *Pseudomonas putida* | TCH-3 | 9.407137 | -0.83702 | PRJEB38443 | Unicycler | ERZ1426505 | 1 | 5,995,929 | 62.53 | 5,995,929 |
| BB1459 | ERS4590906 | *Citrobacter werkmanii* | TCH-3 | 9.407137 | -0.83702 | PRJEB38443 | Unicycler | ERZ1416199 | 14 | 6,073,328 | 51.84 | 5,294,888 |
| BB1460 | ERS4590907 | *Citrobacter werkmanii* | TCH-3 | 9.407137 | -0.83702 | PRJEB38443 | Unicycler | ERZ1416201 | 9 | 6,065,016 | 51.83 | 5,266,939 |
| BB1491 | ERS4590908 | *Citrobacter werkmanii* | TCH-3 | 9.407137 | -0.83702 | PRJEB38443 | Unicycler | ERZ1416203 | 11 | 6,091,743 | 51.84 | 5,293,391 |
| BB1462 | ERS4590909 | *Pseudomonas stutzeri* | TTH-1 | 9.394171 | -0.822951 | PRJEB38443 | Unicycler | ERZ1416159 | 2 | 4,556,022 | 63.88 | 4,496,755 |
| BB1465 | ERS4590912 | *Klebsiella pneumoniae* | TTH-1 | 9.394171 | -0.822951 | PRJEB38443 | Unicycler | ERZ1416162 | 18 | 5,993,594 | 56.51 | 5,232,240 |
| BB1466 | ERS4590913 | *Citrobacter werkmanii* | TTH-1 | 9.394171 | -0.822951 | PRJEB38443 | Unicycler | ERZ1416164 | 25 | 6,073,805 | 51.83 | 5,245,072 |
| BB1467 | ERS4590914 | *Providencia rettgeri* | TTH-1 | 9.394171 | -0.822951 | PRJEB38443 | Unicycler | ERZ1416166 | 4 | 4,539,198 | 40.51 | 4,364,935 |
| BB1468 | ERS4590915 | *Citrobacter youngae* | TTH-1 | 9.394171 | -0.822951 | PRJEB38443 | Unicycler | ERZ1416168 | 5 | 5,477,708 | 51.47 | 4,798,981 |
| BB1471 | ERS4590918 | *Escherichia coli* | TTH-2 | 9.39392 | -0.822843 | PRJEB38443 | Unicycler | ERZ1416170 | 17 | 5,571,584 | 50.81 | 4,889,620 |
| BB1472 | ERS4590919 | *Citrobacter werkmanii* | TTH-2 | 9.39392 | -0.822843 | PRJEB38443 | Unicycler | ERZ1416172 | 9 | 6,025,432 | 51.83 | 5,254,171 |
| BB1473 | ERS4590920 | *Citrobacter werkmanii* | TTH-2 | 9.39392 | -0.822843 | PRJEB38443 | Unicycler | ERZ1416174 | 14 | 6,090,333 | 51.83 | 5,293,374 |
| BB1474 | ERS4590921 | *Citrobacter werkmanii* | TTH-2 | 9.39392 | -0.822843 | PRJEB38443 | Unicycler | ERZ1416176 | 26 | 6,127,255 | 51.84 | 5,293,350 |
| BB1475 | ERS4590922 | *Pseudomonas aeruginosa* | TTH-3 | 9.392537 | -0.819811 | PRJEB38443 | Unicycler | ERZ1416179 | 21 | 6,958,305 | 66.04 | 2,060,458 |
| BB1478 | ERS4590925 | *Citrobacter werkmanii* | TTH-3 | 9.392537 | -0.819811 | PRJEB38443 | Unicycler | ERZ1416181 | 20 | 6,083,948 | 51.84 | 5,293,353 |
| BB1479 | ERS4590926 | *Citrobacter werkmanii* | TTH-3 | 9.392537 | -0.819811 | PRJEB38443 | Unicycler | ERZ1416183 | 10 | 6,087,453 | 51.83 | 5,294,797 |
| BB1480 | ERS4590927 | *Citrobacter werkmanii* | TTH-3 | 9.392537 | -0.819811 | PRJEB38443 | Unicycler | ERZ1416185 | 10 | 6,087,123 | 51.83 | 5,293,032 |
| BB1484 | ERS4590931 | *Pseudomonas putida* | TWH-2 | 9.40099 | -0.850922 | PRJEB38443 | Unicycler | ERZ1416187 | 5 | 5,733,631 | 62.4 | 5,571,987 |
| BB1486 | ERS4590933 | *Comamonas aquatica* | TWH-2 | 9.40099 | -0.850922 | PRJEB38443 | Unicycler | ERZ1416189 | 4 | 4,069,967 | 64.48 | 4,066,208 |
| BB1487 | ERS4590934 | *Providencia rettgeri* | TWH-2 | 9.40099 | -0.850922 | PRJEB38443 | Unicycler | ERZ1416191 | 7 | 5,003,304 | 40.82 | 4,673,756 |
| BB1490 | ERS4590937 | *Citrobacter werkmanii* | UWTP-2 | 9.444945 | -0.757774 | PRJEB38443 | Unicycler | ERZ1416193 | 10 | 6,085,532 | 51.83 | 5,293,555 |
