# Supplementary material for: Dissemination Routes of Carbapenem and Pan-Aminoglycoside Resistance Mechanisms in Hospital and Urban Wastewater Canalizations of Ghana
Source: mSystems. 2022 Feb 1;7(1):e01019-21. doi: 10.1128/msystems.01019-21 (PMC8805638; doi:10.1128/msystems.01019-21)
Supplement: TABLE S6 [file msystems.01019-21-st006.docx]

**Table S6**

| Plasmid | Plasmid type | Size (bp) | %GC | Isolate | ENA sample | Host species | Source | Latitude | Longitude | ENA project | ENA analysis |
| --- | --- | --- | --- | --- | --- | --- | --- | --- | --- | --- | --- |
| pPS-VIM-5 | Novel | 59,267 | 58.0 | BB1462 | ERS4590909 | *Pseudomonas stutzeri* | TTH-1 | 9.394171 | -0.822951 | PRJEB38443 | ERZ1458876 |
| pPP-DIM-1 | Novel | 69,823 | 60.7 | BB1484 | ERS4590931 | *Pseudomonas putida* | TWH-2 | 9.40099 | -0.850922 | PRJEB38443 | ERZ1458873 |
| pPR-NDM-1A | pPrY2001-like | 110,068 | 41.6 | BB1467 | ERS4590914 | *Providencia rettgeri* | TTH-1 | 9.394171 | -0.822951 | PRJEB38443 | ERZ1458874 |
| pPR-NDM-1B | pPrY2001-like | 210,081 | 47.0 | BB1487 | ERS4590934 | *Providencia rettgeri* | TWH-2 | 9.40099 | -0.850922 | PRJEB38443 | ERZ1458875 |
| pCW-NDM-1 | IncC | 161,908 | 50.9 | BB1472 | ERS4590919 | *Citrobacter werkmanii* | TTH-2 | 9.39392 | -0.822843 | PRJEB38443 | ERZ1458879 |
| pCY-NDM-1 | IncC | 252,108 | 50.7 | BB1468 | ERS4590915 | *Citrobacter youngae* | TTH-1 | 9.394171 | -0.822951 | PRJEB38443 | ERZ4172607 |
| plasmoidNDM-1 | Non-typable | 9,301 | 50.9 | BB1471 | ERS4590918 | *Escherichia coli* | TTH-2 | 9.39392 | -0.822843 | PRJEB38443 | ERZ1458884 |
| pCW-CTX-M-15A | pKPC-CAV1321-like | 258,548 | 50.0 | BB1479 | ERS4590926 | *Citrobacter werkmanii* | TTH-3 | 9.392537 | -0.819811 | PRJEB38443 | ERZ1458877 |
| pCW-CTX-M-15B | pKPC-CAV1321-like/IncC | 399,467 | 50.4 | BB1459 | ERS4590906 | *Citrobacter werkmanii* | TCH-3 | 9.407137 | -0.83702 | PRJEB38443 | ERZ1458878 |
| pCY-CTX-M-15 | IncHI1B/IncFIB  (pNDM-MAR-like) | 362,852 | 47.5 | BB1468 | ERS4590915 | *Citrobacter youngae* | TTH-1 | 9.394171 | -0.822951 | PRJEB38443 | ERZ4172607 |
| pCY-OXA-1 | IncR | 54,473 | 54.0 | BB1468 | ERS4590915 | *Citrobacter youngae* | TTH-1 | 9.394171 | -0.822951 | PRJEB38443 | ERZ4172607 |
| pKP-CTX-M-15 | IncHI1B/IncFIB/IncC  (pNDM-MAR-like/IncC) | 395,796 | 47.9 | BB1465 | ERS4590912 | *Klebsiella pneumoniae* | TTH-1 | 9.394171 | -0.822951 | PRJEB38443 | ERZ1458883 |
